# Supplementary material for: Role and Regulatory Mechanism of circRNA_14820 in the Proliferation and Differentiation of Goat Skeletal Muscle Satellite Cells
Source: Int J Mol Sci. 2024 Aug 15;25(16):8900. doi: 10.3390/ijms25168900 (PMC11354305; doi:10.3390/ijms25168900)
Supplement: Supplementary file 1 [file ijms-25-08900-s001.zip › ijms-3121810-supplementary.pdf]

Supplementary information

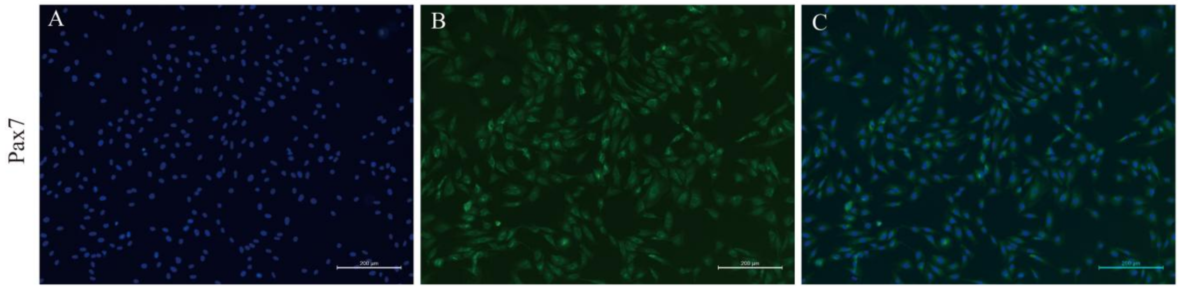

Supplementary Figure S1. Immunofluorescence identification of SMSCs in goats. A, DAPI nuclear staining of cells in the experimental group. B, immunofluorescence of Pax7 antibody in the experimental group. C, DAPI/Pax7 in the experimental group.

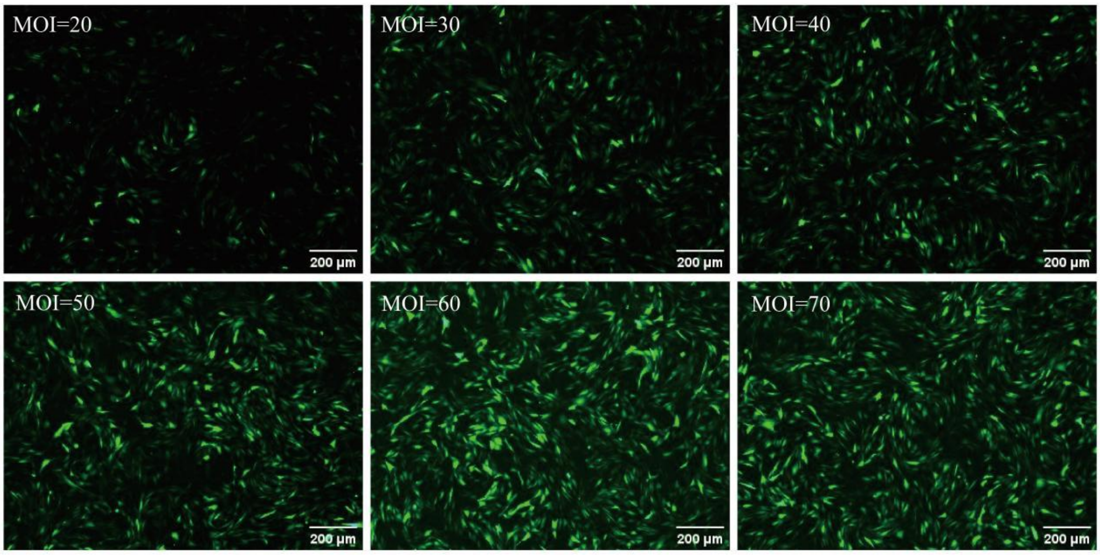

Supplementary Figure S2. Screening of complex MOI values for circ_14820 overexpressed lentivirus infection.

Supplementary Table S1. Information of primers used in this study.

Primer Name	Sequence of Primer (5′-3′)

circ_14820 (Divergent)	F:CCTAGTAAAGAAGAACGGACACAGT<br>R:CAATCGATATCTTGGTCTCGCAGG
circ_14820 (Convergent)	F:ACCTCGTCATCAGAGAAAGCC<br>R:CTTGGTCCCGCAGAAGACAA
GADPH	F:GCAAGTTCCACGGCACAG<br>R:GGTTCACGCCCATCACAA
U6	F:GGAACGATACAGAGAAGATTAGC<br>R:TGGAACGCTTCACGAATTTGCG
18S mRNA	F:ATGCCAGAGTCTCGTTCGTTAT<br>R:CGGACAGGATTGACAGATTGAT
MyoG	F:GGACCCTACAGATGCCCACA<br>R:TTGGTATGGTTTCATCTGGG
MYHC	F:CCACATCTTCTCCATCTCTG<br>R:GGTTCCTCCTTCTTCTTCTC
MyoD	F:GTGCAAACGCAAGACGACTA<br>R:GCTGGTTTGGGTTGCTAGAC
Myf5	F:CGGTGTCTCCCCTATCTATCTC<br>R:ATGCAGGAGCCGTCGTAGAAGT
PAX7	F:AGGACGAAGCGGACAAGAA<br>R:TCCAGACGGTTCCCTTTGT
CCND1	F:TCTCCTATCACCGCCTGACA<br>R:TTGGGGTCCAAGTTCTGCTG
CCND2	F:CCGTCGATGATTGCAACTGG<br>R:GGCAATCCACATCCGTGTTG
miR-206	CCGTGGAATGTAAGGAAGTGTGTGG
Novel-miRNA 6_9154	CGCTAAGTGCTTCCATGTTTTAGTGA

A

B

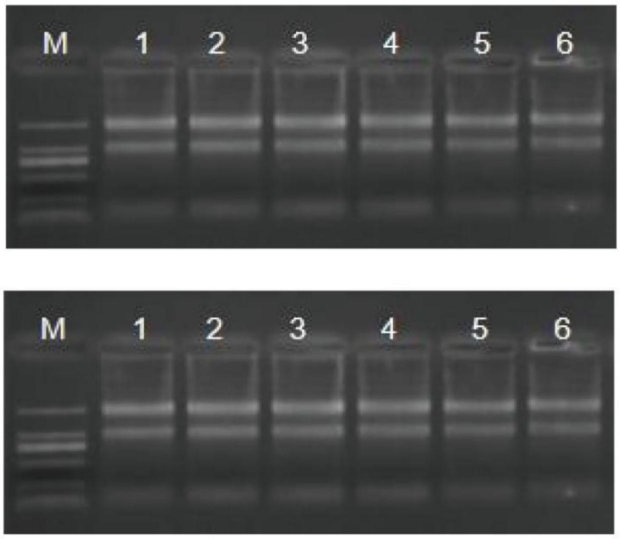

Supplementary Figure S3. RNA quality testing. A, RNA quality testing for small RNA sequencing.B. RNA quality testing for mRNA sequencing.

Sample	RawReads	RawBases	CleanReads	CleanBases	CleanRatio	Q20	Q30	GC
circ_14820_1	44067782	2203389100	28156851	679119340	63.89 %	99.22 %	97.21 %	47.55 %
circ_14820_2	34738191	1736909550	24069377	585591606	69.29 %	99.14 %	96.97 %	47.62 %
circ_14820_3	37274108	1863705400	25863417	629275632	69.39 %	99.23 %	97.20 %	47.68 %
control_1	50726045	2536302250	35980439	880235252	70.93 %	99.25 %	97.33 %	47.35 %
control_2	35954065	1797703250	23910204	583226957	66.50 %	99.29 %	97.49 %	47.78 %
control_3	47247592	2362379600	34558938	845349470	73.14 %	99.25 %	97.30 %	47.44 %

Supplementary Table S2. Statistics of cleandate data from small RNA sequencing

Supplementary Table S3. Genome mapping results of clean reads obtained by small RNA sequencing

Supplementary Table S4. Statistics of cleandate data from mRNA sequencing

Sample	RawReads	RawBases	CleanReads	CleanBases	CleanRatio	Q20	Q30	GC
circ_14820_1	75390094	11308514100	74882004	11205349682	99.33 %	98.21 %	94.66 %	48.31 %
circ_14820_2	77735082	11660262300	77294154	11554685724	99.43 %	98.32 %	94.93 %	48.27 %
circ_14820_3	97667554	14650133100	97088704	14530494581	99.41 %	98.21 %	94.67 %	48.18 %
control_1	97425502	14613825300	96826704	14500124078	99.39 %	98.20 %	94.70 %	48.70 %
control_2	49422268	7413340200	48909040	7316892918	98.96 %	98.03 %	94.34 %	50.05 %
control_3	37608696	5641304400	37263598	5575752641	99.08 %	98.21 %	94.80 %	50.20 %

sample	totalReads	mappedReads	mapRate	uniq	paired	single	selfANDmate	mapDiffCHR
circ_14820_1	74882004	61433126	82.04 %	58751002	57510056	1306736	60126390	132538
circ_14820_2	77294154	63667504	82.37 %	60780400	59393558	1464046	62203458	198012
circ_14820_3	97088704	80134074	82.54 %	76517981	74821368	1791594	78342480	221688
control_1	96826704	75123415	77.59 %	71924185	70422106	1581065	73542350	145024
control_2	48909040	30821729	63.02 %	29603997	28126570	1506473	29315256	62444
control_3	37263598	23902109	64.14 %	22957389	21801506	1177423	22724686	49966

Supplementary Table S5. Genome mapping results of clean reads obtained by mRNA sequencing

Sample	Total sRNA	Mapped sRNA
circ_14820_1	325142	188635(58.02 %)
circ_14820_2	288674	163291(56.57 %)
circ_14820_3	295210	169869(57.54 %)
control_1	383867	223952(58.34 %)
control_2	280263	162285(57.90 %)
control_3	372237	218326(58.65 %)
